# Comparative assessment of Texas horned lizard (*Phrynosoma cornutum*) gut microbiome diversity and composition throughout transition from captivity to wild

**DOI:** 10.3389/frmbi.2025.1601442

**Published:** 2025-06-18

**Authors:** Cameron R. Forehand, Sierra N. Smith, Forrest Nielsen, Blake Bauer, Jessa L. Watters, Ray W. Moody, Daniel J. Becker, Hayley Lanier, Katharine Marske, Cameron Siler

**Affiliations:** ^1^ Department of Herpetology, Sam Noble Oklahoma Museum of Natural History, Norman, OK, United States; ^2^ School of Biological Sciences, University of Oklahoma, Norman, OK, United States; ^3^ Department of Biology, University of Texas Arlington, Arlington, OK, United States; ^4^ Department of Herpetology, Oklahoma City Zoo, Oklahoma City, OK, United States; ^5^ Oklahoma Department of Transportation, Environmental Programs Division, Natural Resources Program, University of Oklahoma, Norman, OK, United States; ^6^ Natural Resources Program, Tinker Air Force Base, Oklahoma City, OK, United States

**Keywords:** captivity, gut microbiome, headstarting, reptile, 16S rRNA

## Abstract

Microbiomes play a key role in the health of animal hosts. To improve conservation translocation programs like headstarting, it is necessary to consider how the structure of these programs impact the host-associated microbiome. Bringing animals into captivity introduces novel diets and environments; however, the extent to which these factors contribute to the structure of the host’s gut microbiome remains poorly understood. Additionally, it is unclear if periods of captivity leave a lasting signature on the host-associated gut microbiome, which could impact individual health and fitness in the long-term. In this study, we repeatedly sampled the gut microbiome of a cohort of headstart Texas horned lizards (*Phrynosoma cornutum*) throughout their transition from captivity to the wild. We also collected samples of extrinsic microbial communities present in their captive and wild diet and environment. Finally, we sampled the gut microbiome of wild resident lizards to serve as a baseline comparison. Using 16S rRNA microbial inventories, we examined differences in microbial community composition and diversity between pre-release headstart, post-release headstart, and resident lizards of the wild population. Additionally, we assessed the contribution of environmental and dietary microbial communities to the assembly of *P. cornutum* gut microbiomes in captivity and the wild. Our results suggest captive *P. cornutum* harbor gut microbiomes that are distinct from their wild counterparts. However, within two-months post-release, the headstart gut microbiome restructures to be indistinguishable from the wild resident microbiome. Microbiomes associated with the captive diet and environment are distinct in beta diversity, but not alpha diversity, from those in the wild. Our results provide important insights into host-associated microbiome dynamics associated with transition from captivity to the wild and can be used to inform conservation translocation practices.

## 1 Introduction

Host-associated microbial communities residing within different parts of the body and their importance for promoting host fitness is well established (Berg et al., 2020). Extensive studies in humans, as a model organism, and in domestic animals has revealed a close connection between symbiotic microbial communities and essential host functions such as nutrient extraction, metabolism, immune development, behavior, and reproduction (Knight et al., 2017; Metcalf et al., 2017; Wang et al., 2017; Carranco et al., 2022; Couch and Epps, 2022). Furthermore, the continued development and advancement of next-generation sequencing methods has made DNA sequencing faster, easier, and more accessible, enabling in-depth exploration of microbial communities in novel systems (Couch and Epps, 2022; Combrink et al., 2023). In recent years, there has been a burgeoning field of research on host-associated microbiomes in wild animal populations, with a particular focus on endangered and threatened species (West et al., 2019).

Early wildlife microbiome studies compared host-associated microbiome composition and diversity across captive and wild environments (West et al., 2019; Zhu et al., 2021). It quickly became clear that these communities vary across conspecific captive and wild populations, often exhibiting structural differences. Many researchers hypothesized this was due to the influence of extrinsic environmental factors (i.e. habitat, diet, human handling activities, use of supplements and/or antibiotics, varying degrees of contact with conspecifics; Kohl et al., 2017; Diaz and Reese, 2021; Dallas and Warne, 2022). Despite previous findings suggesting that captivity alters the host-associated microbiome community membership and composition regardless of taxa, it does not exhibit nearly as universal of an effect on alpha diversity (Diaz and Reese, 2021). Instead, changes are highly species specific; for example, the crocodile lizard (*Shinisaurus crocodilurus*), pangolin (*Manis javanica*), and elephant seal (*Mirounga leonina*) demonstrate increased alpha diversity in captivity, while the red panda (*Ailurus fulgens*), brown kiwi (*Apteryx mantelli*), and red-shanked douc (*Pygathrix nemaeus*) show decreased diversity (Nelson et al., 2013; Kong et al., 2014; Clayton et al., 2016; Tang et al., 2020; Diaz and Reese, 2021; San Juan et al., 2021; Yan et al., 2021). In contrast, studies on the Dybowski’s brown frog (*Rana dybowskii*), green turtle (*Chelonia mydas*), and turkey (*Meleagris gallopav*) report no difference between captive and wild microbiomes (Scupham et al., 2008; Campos et al., 2018; Tong et al., 2019; Diaz and Reese, 2021).

Even if most wildlife host-associated microbiomes change in captivity, it is unclear if it ultimately impacts their fitness and health (Diaz and Reese, 2021). Some studies argue that these changes may benefit the host in captivity, such as different bacterial taxa shown to be better suited to helping digest a captive versus wild diet, leading to changes in community composition (Kohl et al., 2014; Diaz and Reese, 2021). However, others suggest shifts might indicate a state of dysbiosis, or disruption to the host-associated microbial community and its functions, which often coincides with disease or environmental stress and may result in decreased fitness (Amato et al., 2016; Trevelline et al., 2019; West et al., 2019). Furthermore, potentially pathogenic bacteria have been documented in captive populations, including observations of *Brachybacterium* sp., *Brevibacterium* sp., and *Nesterenkonia* spp. present in a captive headstart population of Fijian crested iguanas (*Brachylophys vitiensis*), which have been shown to cause bloodstream and other infections in human hosts (Gruner et al., 1993; Tamai et al., 2018; Eliades et al., 2021). To better understand the relationship between host-associated microbiome changes in captivity and individual fitness and survivorship, longitudinal studies are necessary to monitor these shifts and test for correlates with other health metrics or survivorship.

Presently, few studies have characterized temporal changes in the host-associated microbiomes of individuals transitioning between captivity and the wild (Diaz and Reese, 2021; Korpita et al., 2023). However, findings from previous studies on the Tasmanian devil (*Sarcophilus harrisii*), deer mouse (*Peromyscus maniculatus*), and boreal toad (*Anaxyrus boreas*) demonstrate that the host-associated microbiomes of reintroduced animals resemble those of wild counterparts within weeks (Chong et al., 2019; Schmidt et al., 2019; Korpita et al., 2023). In contrast, research on the white-footed mouse (*Peromyscus leucopus*) and giant panda (*Ailuropoda melanoleuca*) found that captivity could have a more lasting signature on translocated individuals’ gut microbiomes (Yao et al., 2019; Leeuwen et al., 2020). Captive white-footed mice fed a more natural unprocessed diet shared more microbiota with wild mice than those fed on dry standardized pellets only (Leeuwen et al., 2020). Further, it took the gut microbiome of reintroduced pandas up to one year to be fully stabilized and resilient to pathogen invasion (Yao et al., 2019). Given the importance of microbiomes for animal health, evaluating and monitoring changes to microbiome community membership and diversity wildlife reintroductions remains essential, especially for threatened and endangered species.

If changes in the host-associated microbiome of released individuals impact overall fitness, conservation practitioners should consider these microbial communities in their management plans (Bahrndorff et al., 2016; Eliades et al., 2021). A common translocation program utilized by wildlife managers to bolster populations of threatened and endangered species is headstarting—wild neonates are brought into captivity to be raised past the most vulnerable life stages, thereby increasing the survivorship of early age classes (Mack et al., 2018; Brown et al., 2021; Eliades et al., 2021). Headstarted individuals are then reintroduced as juveniles or young adults, which helps to bolster existing population size. To contribute to a small but growing body of literature on host-associated microbial community changes of translocated individuals, we collaborated with a headstarting initiative for Texas horned lizards (*Phrynosoma cornutum*) at the Oklahoma City Zoo and Botanical Garden in northeast Oklahoma City (Oklahoma County), Oklahoma USA to conduct a temporal assessment of changes in the gut microbiome of headstarted hosts.

*Phrynosoma cornutum* is a reptile species of the American Southwest, with a range spanning a broad portion of the south-central US and extending south into Mexico (Sherbrooke, 2003; Vesy et al., 2021). In recent years, Texas horned lizards have faced population decline due to largely anthropogenic factors; increased urbanization throughout their range has led to habitat loss, fragmentation, and degradation (Audsley et al., 2006; Vesy et al., 2021). Additionally, the introduction of the red imported fire ant (*Solenopsis invicta*), in addition to widespread pesticide use, has also contributed to the loss of their preferred prey item, the harvester ant (*Pogonomyrmex* spp.; Donaldson et al., 1994; Robertson and Bossart, 2024). As a result, the species continues to experience population declines and localized extirpation, and is considered threatened in Texas, a species of special/conservation concern in Missouri and Colorado, and a species of greatest conservation need in Oklahoma (Vesy et al., 2021; Missouri Department of Conservation, 2024; Colorado Parks & Wildlife, 2025; Oklahoma Department of Wildlife Conservation, 2025; Texas Parks and Wildlife, 2025).

To address continued population declines within the state, the Oklahoma City Zoo and Botanical Garden (OKC Zoo) established a headstart program in 2019. The initiative works in collaboration with the natural resource department at Tinker Air Force Base (TAFB) in Oklahoma County, Oklahoma USA. A naturally occurring population of *P. cornutum* inhabits Wildlife Reserve 3 (WR3) of TAFB. The OKC Zoo collects clutches and hatchlings from WR3 to raise in captivity for a 1 or 2-year period. Individuals are then reintroduced to WR3 to counteract high hatchling mortality rates and bolster population numbers (Sacerdote-Velat et al., 2014). Precise tracking of wild residents and released headstarts with very high frequency radio-transmitters and harmonic radar offers a unique opportunity to closely monitor changes in the host-associated microbiome of reintroduced lizards and make comparisons to wild conspecifics.

In this study, we collected samples of headstart *P. cornutum* gut microbiomes for four months prior to their release, and three months following their release. After the headstart lizards were reintroduced, we also collected samples of wild resident lizards for direct comparison. To better understand the factors driving host-microbiome variation across captive and wild settings, we sampled microbial communities present in lizard diets and environments at the OKC Zoo and TAFB. We provide an assessment and summary of changes in the community composition and diversity of the *P. cornutum* gut microbiome across captive and wild settings to 1) determine if/how the host-associated gut microbiome is affected by host translocation, and 2) compare the host-associated gut microbiome of translocated and resident lizards. These results provide insight into extrinsic factors driving changes in host-associated microbiomes and contribute to a better understanding of how broad environmental change may impact host-associated microbiomes. As the longevity and security of wildlife populations around the globe are increasingly threatened by global change, conservation practitioners need better insight into the role of the host-associated microbiome in wildlife health and fitness to make more informed management decisions.

## 2 Materials and methods

### 2.1 Captive husbandry

Wild hatchling lizards were collected for the headstart program from August to September 2021 from a naturally occurring population of *P. cornutum* present on WR3 of TAFB. The cohort of 18 lizards was raised in captivity at the designated headstart facilities (“Lizard Lab”) at the OKC Zoo from August/September 2021 to May 2023. During this period, two individuals were euthanized by OKC Zoo Veterinary staff due to health issues. While at the Lizard Lab, headstart lizards were housed individually in 20 gallon-long glass aquariums with approximately two inches of substrate consisting of a mixture of sand, mulch, decomposed granite, and locally sourced soil. The aquariums were decorated with rock structures and cholla wood for refuge and basking. A Mistking misting system was programmed to provide water twice daily (Item #309426, Mistking, Jungle Hobbies Ltd., Emeryville, ON, CA). Lizards were fed a combination of insects including fruit flies (*Drosophila hydei* primarily, some *D. melanogaster*), crickets (*Acheta domesticus*), and mealworms (*Tenebrio molitor*) once a day. Before feeding, all insects were dusted with a Repashy Formic-Cal Plus AntEater supplement (Item #TREP2298S, Repashy Ventures Inc., Oceanside, CA, USA). Prey items fed to lizards were primarily sourced from the online retailer Josh’s Frogs, LCC or from OKC Zoo stocks.

On May 5^th^, 2023, a cohort of 16 headstart lizards (seven females, nine males) were released to the population on WR3. The release date coincided with the *P. cornutum* breeding season, which takes place from late April through mid-June (Ballinger, 1974). All tracked resident and headstart lizards were considered adults and could have been reproductively active. Initially, all 16 headstart individuals were placed together in an outdoor enclosure for a three-week, soft release period before being fully released to the reserve. The enclosure was an eight m by nine m pen, consisting of 36 cm vinyl flashing dug approximately 13 cm into the ground. Deer fencing was placed over the top of the pen to exclude predators, while prey items had access through holes drilled in the vinyl flashing. The enclosure was placed in an area of WR3 occupied by the resident population. All headstart and wild resident lizards were tagged with one of three monitoring devices to track survivorship and to locate for gut microbiome resampling: a 0.03 g diode tag for detection via harmonic radar (RECCO Rescue Systems, Lidingo, Sweden; Vesy et al., 2021), a 1.0 g very high frequency radio-transmitter (BD-2 series, Holohil Systems, Carp, ON, CA; Vesy et al., 2021), or a 1.0 g digital radio tag (HybridTag, Cellular Tracking Technology, Rio Grande NJ, USA). Lizards weighing ≤10g were placed on a harmonic radar tag to ensure that tags did not exceed 10% of body mass. Additional details on the headstart program at the OKC Zoo can be reviewed in Barrett et al. (2022).

### 2.2 Microbial community sampling

To assess the contributions of diet and environment on headstarted *P. cornutum* gut microbiomes during transitions from captivity to wild release, we collected samples of all of these groups from January to July 2023.For a baseline comparison with reintroduced lizards, we also sampled the gut microbiome of the wild, resident population on WR3 from May to July 2023. We wore sterile gloves during sample collection and immediately placed all samples into a 1.5 mL cryogenic vial with 1 mL Zymo DNA/RNA Shield (Item #R1100, Zymo Research Products, Irvine, CA, USA) and stored at -20°C until DNA extraction.

#### 2.2.1 Gut microbiome sampling

To evaluate the headstart lizard gut microbiome, we collected gut microbiome samples of all 16 headstart lizards twice a month from January to April 2023, and once a month from May to July 2023. We additionally aimed to sample all tracked resident lizards (ranging from 13–19 individuals) once a month from May to July 2023. The number of tracked wild lizards fluctuated per month with the discovery of new individuals, lizard mortalities, and VHF tag failures leading to censored individuals. Gut microbiome samples were collected by gently inserting a sterile mini-tip polyester swab (REF #25-1000 1PD, Puritan Medical Products, Guilford, ME, USA) into the cloaca and rotating it 3–4 times. Cloacal swabs are an established and effective method to sample the gut microbiomes of lizards (Colston, 2017; Smith et al., 2021; Bunker et al., 2022). We extracted total microbial DNA from a subset of 169 headstart and 40 resident gut microbiome samples.

#### 2.2.2 Environmental sampling

To assess the contribution of the environmental microbiome to the headstart lizard gut microbiome, from January to April 2023, we collected 10 swab samples of the microbiota present in the headstart lizard’s enclosures each month. We used a random number generator to randomly select 10 enclosures to sample. We also sampled the water from the Mistking misting system in each selected enclosure (Item #309426, Mistking, Essex, ON, CA). Once the headstart lizards were translocated to WR3, from May to July 2023, we collected soil microbiome samples where headstart lizards were relocated. We collected at least 10 soil samples a month. To sample microbiota present in the lizards’ environment, we rubbed a sterile polyester swab (REF #25-806 1PR, Puritan Medical Products, Guilford, ME, USA) across the surface of the cage substrate or native soil for 5–10 seconds. To sample water from the enclosure misting system, we inserted a sterile polyester swab (REF #25-806 1PR, Puritan Medical Products, Guilford, ME, USA) into the stream of mist and rotated it for 5–10 seconds until the swab was well-saturated with water. We extracted total microbial DNA from a subset of 82 environmental samples.

#### 2.2.3 Dietary sampling

To assess the contribution of dietary microbiota to the headstart lizard gut microbiome, we sampled microbial communities associated with prey items. From January to April 2023, while the lizards were housed in captivity, we collected 10 samples per month of each prey item fed to the lizards. We also collected 10 samples per month of the Repashy Formic-Cal Plus Ant-Eater-supplement. Whole insects were collected wearing sterile gloves. We sampled the Repashy Formic-Cal Plus Ant Eater supplement by introducing a sterile polyester swab (REF #25-806 1PR, Puritan Medical Products, Guilford, ME, USA) into the supplement bottle and swirling it throughout the bottle for 3–4 seconds.

After the headstarted lizards were released, four insect pitfall traps were placed in areas of high lizard activity to capture the diversity of possible prey items at WR3. *P. cornutum* are generally considered harvester ant (*Pogonomyrmex* spp.) specialists, but there are currently no known populations of harvester ant on WR3 (Ramakrishnan et al., 2018). Instead, resident lizards of WR3 have been shown to primarily consume other ant genera such as *Pheidole* and *Formica* (Ramakrishnan et al., 2018). Additionally, other wild populations of *P. cornutum* have been shown to predate other non-ant arthropods (Heuring et al., 2019; Richards and Watson, 2024). To sample the total possible diversity, we collected all arthropods present in traps. The traps consisted of small plastic cups placed approximately 4 inches deep into the ground and filled to one inch with a mixture of dish soap and water. Insect traps were checked once or twice a week approximately 48–72 hours after setting. If a lizard was located near a likely prey item, or observed eating, we opportunistically collected those prey items. Potential prey items included Coleoptera (beetles), Araneae (spiders), Orthoptera (grasshoppers/crickets), Diptera (flies), and Hymenoptera (ants) and were collected with sterile forceps and then washed with ethanol. We extracted total microbial DNA from a subset of 65 diet samples.

### 2.3 DNA extractions

We extracted total microbial DNA from 209 lizard gut microbiome samples, 82 environmental samples, and 65 diet samples using the ZymoBIOMICS DNA Miniprep Kit (Item #D4300T, Zymo Research Products, Irvine, CA, USA). For each DNA extraction batch, we included a blank sample to serve as a negative control. We also included two microbial community standard samples which consisted of a known community of microorganisms (Item #D6300, Zymo Research Products, Irvine, CA, USA; **Supplementary Figure 1**) in an extraction batch to serve as a positive control.

All swab samples (gut microbiome and environmental samples) were extracted following standardized kit protocols. Additional steps were taken to extract whole insect samples. First, individual insect specimens were removed from DNA/RNA Shield and cut into small pieces using a sterile razor blade on a sterile surface (Watson et al., 2023). Each insect sample was then shaken on a vortex mixer for two 20 minute periods; first, using a lysis tube with larger 0.1 and 2.0 mm beads (Item #S6014, Zymo Research Products, Irvine, CA, USA) to ensure the insect tissue was completely homogenized before being transferred into the standard 0.5 and 0.1 mm BashingBead Lysis Tube (Item #S6012, Zymo Research Products, Irvine, CA, USA) to disrupt the microbial cell walls (Watson et al., 2023).

### 2.4 Library preparation

After extraction, the DNA concentration in each sample was quantified using a Qubit Fluorometer (Item #Q33238, ThermoFisher Scientific, Waltham, Massachusetts, USA). Any lizard gut, diet, or environment samples that did not contain amounts of DNA detectable by the Qubit Fluorometer (<0.05 ng/uL) were excluded from the final sample set used to develop the libraries. Notably, no environmental samples of the captive lizards’ water source contained detectable amounts of DNA. The final libraries included 144 headstart gut microbiome samples, 26 resident gut microbiome samples, 56 environmental samples, and 49 diet samples. Following the protocol described in Kozich et al. (2013), we performed a one-step Polymerase Chain Reaction to amplify the V4 region of the 16S ribosomal RNA (rRNA) gene. Included in the PCR reaction was a sample of a microbial community DNA standard which consisted of DNA extracted and pooled from pure cultures as an additional positive control (Item #D6305, Zymo Research Products, Irvine, CA, USA; **Supplementary Figure 1**).

The PCR product was cleaned with KAPA pure beads at a concentration of 0.6 to remove potential adapter or primer dimer (Item #07983298001, Roche Sequencing, Pleasanton, CA, USA). Before completing the bead clean up protocol, all samples were normalized to 20 uL post-PCR with sterile laboratory-grade water to ensure a consistent KAPA pure bead to sample ratio. The bead cleanup protocol was completed by an Agilent Bravo robot with a 96LT head (Agilent, Santa Clara, CA, USA) at the Oklahoma Medical Research Foundation (OMRF) Consolidated Core Lab. Next, all bead-cleaned PCR products were quantified with a Qubit Fluorometer (ThermoFisher Scientific, Waltham, Massachusetts, USA), normalized to 10 nM of DNA, and pooled into a sterile, 1.5 mL microcentrifuge tube. Pooled libraries were submitted to the OMRF Consolidated Core Lab for 2 x 250 bp paired-end sequencing on an Illumina MiSeq (Illumina, San Diego, CA, USA).

### 2.5 Sequence analysis

Remnant adapter sequences were trimmed using AdapterRemoval v2 with a minimum quality of 30 (Schubert et al., 2016). Sequence data was processed using the QIIME2 microbiome software package (Bolyen et al., 2019). We performed closed reference clustering with a similarity threshold of 99% against the SILVA 138.1 database (Quast et al., 2013) using VSEARCH (Rognes et al., 2016). From our initial dataset consisting of 286 samples, we obtained a total of 2,891,785 sequences that clustered into 32,777 operational taxonomic units (OTUs). First, we filtered out any archaea or nonbacterial sequences (Bolyen et al., 2019; Eliades et al., 2021; Li et al., 2022). We additionally filtered out OTUs that were not present in at least two samples, assuming these features are likely PCR or sequencing errors if only present in one sample. Our dataset was then rarefied to a minimum sequence count of 500 for downstream analysis based upon the Shannon diversity index and OTU richness rarefaction curves (**Supplementary Figures 2, 3**). Of the original dataset, one diet sample and one environment sample were excluded due to poor sequence quality. Some lizards had duplicate gut microbiome samples within one sampling period (biological replicates); we retained only the sample with the greater number of sequences. After all filtering, 214 samples (91 headstart gut microbiome; 20 resident gut microbiome; 55 environmental microbiome; 48 diet microbiome) were used for downstream analysis. To further assess the quality of our data, we analyzed the composition of the positive control community samples against the expected composition (**Supplementary Figure 1**). We successfully identified seven of the eight expected bacterial species. It is important to note we did not identify the presence of *Salmonella enterica* in any of the controls; however, given that we were interested in broadscale shifts in community composition rather than identifying the presence of specific bacterial species, we chose to proceed with the dataset. Raw sequence data from this study can be found in the Sequence Read Archive (SRA) under BioProject PRJNA1218705.

### 2.6 Statistical analysis

To evaluate changes in the gut microbiome of headstart lizards, resident lizards, and microbial communities associated with their diet and environment, alpha diversity (within sample) and beta diversity (among sample) analyses were performed using QIIME 2 (Bolyen et al., 2019). Alpha diversity (Shannon diversity index, OTU richness) and beta diversity (unweighted UniFrac distance, weighted UniFrac distance) metrics were calculated for each sample (Bolyen et al., 2019).

First, we examined the variation in Shannon diversity and OTU richness of the headstart lizard gut microbiome over time. We built linear mixed models in the R software environment with each alpha diversity metric (either Shannon diversity or OTU richness) as the response variable, month of sample collection as a categorical fixed effect, and lizard ID as a random intercept to account for baseline individual variation. We were unable to include random slopes in the model to account for individual variation in response due to the condensed sample sizes post-rarefaction (each individual was missing a representative sample from at least one month). All models were fit with restricted maximum likelihood using the package *lmerTest* (Kuznetsova et al., 2017). We checked model assumptions with QQ plots and assessed statistical significance using the Anova() function in the *stats* package (Chambers and Hastie, 1992). Finally, we utilize the package *emmeans* to obtain pairwise comparisons from our models while adjusting for multiple comparisons with the Tukey method (Lenth et al., 2024).

Further alpha diversity comparisons were completed using the Q2 diversity plugin available through the QIIME2 software, which completes pairwise Kruskal-Wallis tests to determine the statistical significance of group comparisons (Kruskal and Wallis, 1952; Bolyen et al., 2019). We used non-parametric Kruskal-Wallis tests because the residuals did not meet the assumption of normality necessary for ANOVA tests. Each set of analysis was repeated for Shannon Diversity and OTU richness. First, we separately compared the alpha diversity of the gut microbiome of post-release headstart and resident lizards less than one month after release in May 2023, and two months post-release in July 2023. Due to the low number of resident samples post-rarefaction (n = 2), we could not complete alpha diversity comparisons of June samples. Additionally, we choose to complete separate analysis for May and July using Kruskal-Wallis tests, instead of further linear mixed modeling, because few resident individuals had representative samples for both months post-rarefaction (n = 4). Our final set of alpha diversity comparisons again utilized Kruskal-Wallis tests to compare microbial communities present in the *P. cornutum* environment and diet across captivity and the wild.

To assess compositional differences among microbial communities, we utilized two beta diversity indices: unweighted Unifrac and weighted Unifrac distances. Both indices consider phylogenetic distances between observed OTUs, weighted Unifrac distance considers abundance, whereas unweighted Unifrac distance is calculated using the presence or absence of OTUs only (Knight et al., 2018). All beta diversity comparisons were completed using the Q2 diversity plugin available through the QIIME2 software, which completes pairwise PERMANOVAs to determine group significance (Anderson, 2008; Bolyen et al., 2019). Each set of analyses were repeated using unweighted Unifrac and then weighted Unifrac distances. First, to replicate the structure of our alpha diversity analyses, we separately compared the beta diversity of headstart and resident gut microbiome samples less than one month after the release in May 2023 and two months post-release in July 2023. Next, we completed another set of beta diversity analyses including all lizard gut microbiome, diet, and environment samples collected in both captivity and the wild. To best determine broad differences across these diverse microbial communities, we grouped the samples as the following seven categories: 1) headstart gut microbiome pre-release, 2) headstart gut microbiome post-release, 3) resident gut microbiome, 4) diet microbiome pre-release, 5) diet microbiome post-release, 6) environment microbiome pre-release, and 7) environment microbiome post-release. We incorporated these beta diversity data into a principal coordinates analysis (PCoAs) using the QIIME2 software, and we exported the results into R for visualization with the package *ggplot2* (Wickham, 2016).

To assess intra-individual changes in headstart lizard gut microbiomes, we identified the breadth of change (intra-individual unweighted or weighted Unifrac distance) in a headstart individual’s gut microbiome monthly from January 2023 to July 2023. We utilized the Q2 longitudinal plugin available through the QIIME2 software to calculate the beta diversity distance between successive samples collected from the same individual and then visualized these distances with a volatility plot (Bokulich et al., 2018; Bolyen et al., 2019).

## 3 Results

Our initial dataset consisted of 286 samples: 170 lizard gut microbiome samples, 49 prey item microbiome samples, 56 environmental microbiome samples, eight negative samples, two DNA microbial community standards, and one PCR microbial community standard. From our initial dataset, we obtained a total of 2,891,785 sequences that clustered into 32,777 OTUs with a similarity threshold of 99% against the SILVA 138.1 database (Quast et al., 2013). After filtering processes (including rarefying the dataset to a sequence count of 500), we retained 214 samples containing a total 2,644,360 sequences that clustered into 21,366 OTUs (Quast et al., 2013; **Supplementary Table 1**). Raw sequence data from this study can be found in the Sequence Read Archive (SRA) under BioProject PRJNA1218705.

### 3.1 Taxonomic composition of microbial community samples

**Figure 1:**
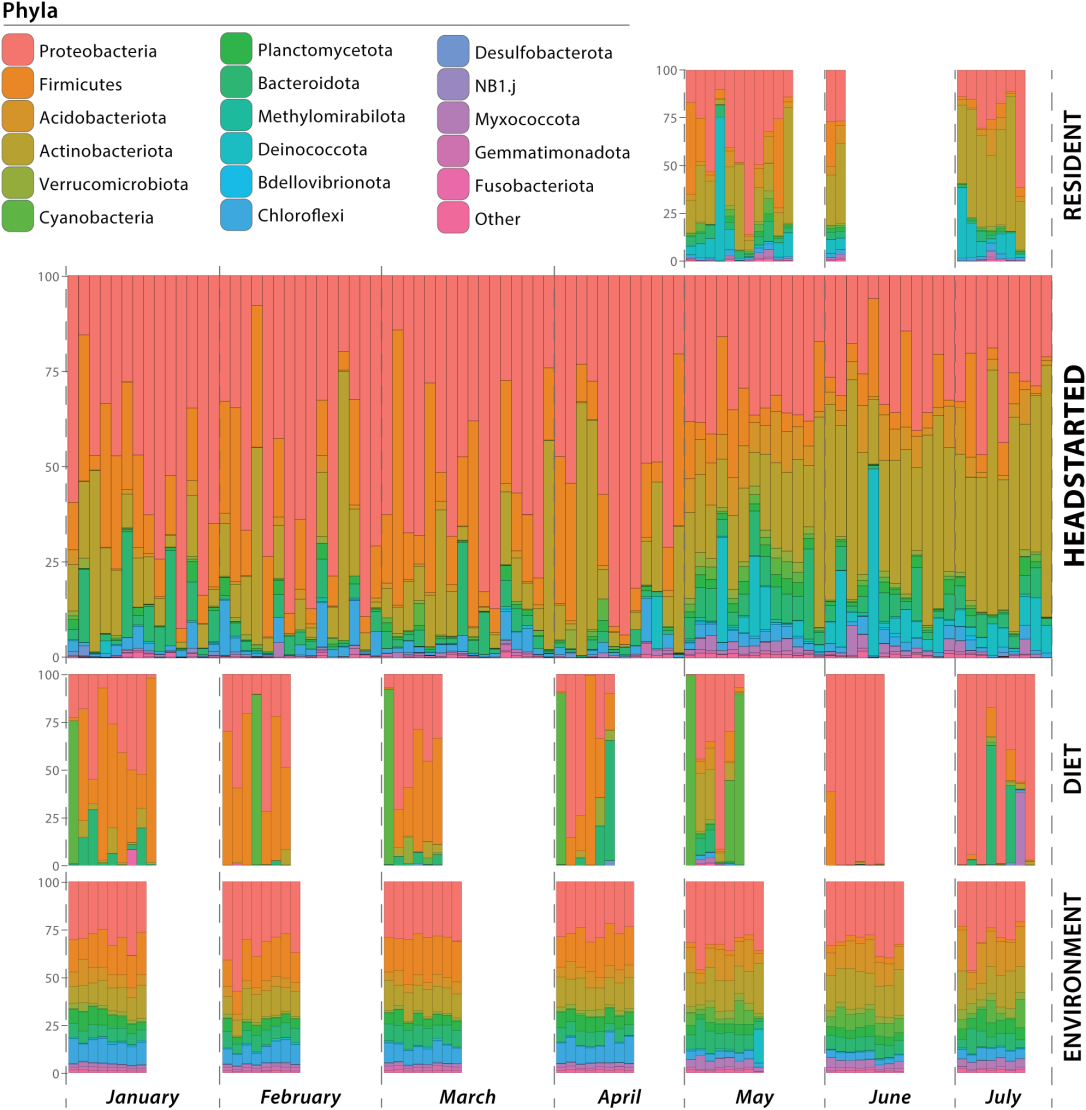
Microbial composition of samples at the phylum level. Samples are grouped by resident lizard gut microbiome, headstart lizard gut microbiome, diet microbial community, and environmental microbial community. Within each group, the samples are ordered temporally. Horizontal bars represent individual samples (i.e. a lizard cloacal swab, environmental substrate swab, or prey item microbiome), and colors correspond to microbial phyla.

While housed in captivity, prior to release, headstart lizard gut microbiomes were composed primarily of the phyla Proteobacteria (average relative abundance of 54.28%), followed by Firmicutes (18.89%) and Actinobacteriota (15.06%). Other dominant phyla (average relative abundance >1%; Suenami et al., 2019; Smith et al., 2023) included Bacteroidota (4.42%) and Chloroflexi (2.80%). After the headstart lizards were released to the wild, Actinobacteriota (32.63%) and Proteobacteria (29.88%) were the most abundant phyla. Other phyla present at an average relative abundance between 1–10% included Acidobacteriota, Bacteroidota, Chloroflexi, Cyanobacteria, Deinococcota, Firmicutes, Myxococcota, Planctomycetota, and Verrucomicrobiota (**Figure 1** **;** **Supplementary Table 2**).

Similarly to post-release headstart gut microbiomes, the phyla Actinobacteriota (average relative abundance of 32.14%) and Proteobacteria (31.45%) were also the most abundant phyla in the wild resident gut microbiomes. Deinococcota (11.58%) and Firmicutes (9.27%) were also prominent phyla, while Acidobacteriota, Bacteroidota, Chloroflexi, Cyanobacteria, Myxococcota, and Planctomycetota were present at average relative abundances between 1–5% (**Figure 1**; **Supplementary Table 2**).

Microbial communities associated with prey items fed to headstart lizards in captivity consisted primarily of the phyla Firmicutes (average relative abundance of 41.13%), followed by Proteobacteria (35.56%) and Cyanobacteria (12.48%). Bacteroidota, Actinobacteriota, and Verrucomicrobiota were present at relative abundances between 1–10%. In the wild, the most abundant phyla associated with prey items was Proteobacteria (68.49%), followed by Cyanobacteria (11.70%). Other phyla present in average relative abundances from 1–10% included Acidobacteriota, Actinobacteriota, Bacteroidota, Firmicutes, and Myxococcota (**Figure 1**; **Supplementary Table 2**).

Microbiomes associated with the captive environment (i.e. headstart lizard enclosures) were dominated by Proteobacteria (30.70%), Firmicutes (18.67%), and Actinobacteriota (13.1%). The phyla Acidobacteriota, Bacteroidota, Chloroflexi, Gemmatimonadota, Myxococcota, Planctomycetota, and Verrucomicrobiota were present at average relative abundances between 1–10%. Similarly to the captive environment, Proteobacteria (32.27%) was the most abundant phyla associated with wild environmental microbiomes. Actinobacteriota (16.79%) and Acidobacteriota (14.01%) also comprised large portions of the wild environmental microbiome, while Bacteroidota, Chloroflexi, Cyanobacteria, Firmicutes, Gemmatimonadota, Myxococcota, Planctomycetota, Verrucomicrobiota were present in relative abundances from 1–10% (**Figure 1**; **Supplementary Table 2**). More detailed taxonomic compositions of samples at the family level are provided in **Supplementary Table 3**.

### 3.2 Alpha and beta diversity analyses

**Figure 2:**
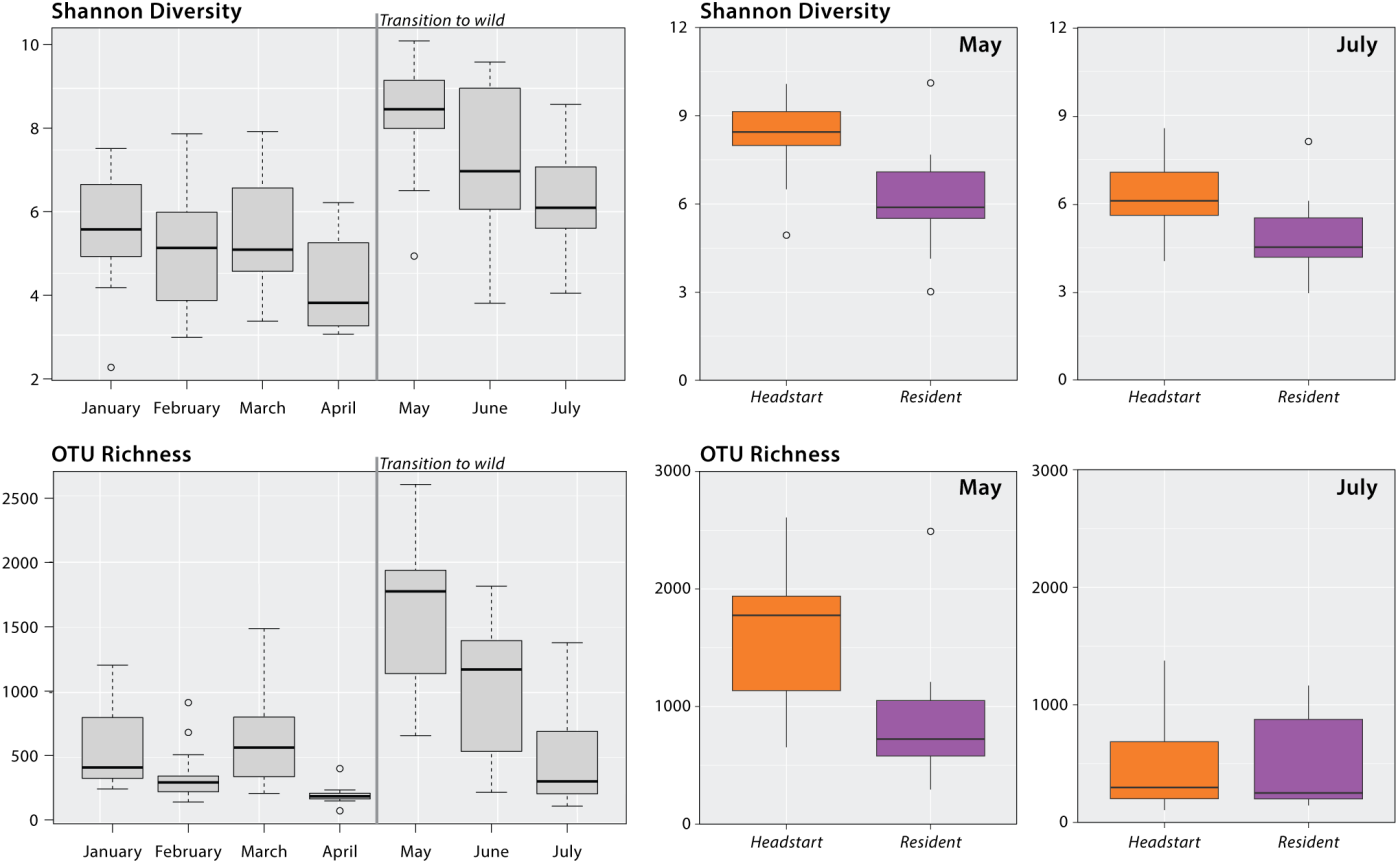
Alpha diversity analysis of headstart and resident lizards. Gray boxplots display the headstart lizard Shannon diversity and OTU richness by month from January to July 2023. Results of linear mixed modeling and *post-hoc* pairwise comparisons can be found in **Supplementary Tables 4, 5**. Colored boxplots display Shannon diversity and OTU richness comparisons of headstart and resident lizard gut microbiomes in May and July 2023. Results of Kruskal-Wallis tests can be found in **Supplementary Table 6**.

Our linear mixed models found a significant effect of month on the Shannon diversity (F(6, 73.88) = 11.482, p < 0.001; **Supplementary Table 4**) and OTU richness (F(6, 74.531) = 21.361, p < 0.001; **Supplementary Table 5**) of headstart lizard gut microbiomes. While in captivity (January–April), the alpha diversity of headstart lizard gut microbiomes was relatively stable, and no significant differences between sampling periods were observed, except for OTU richness in March and April (**Supplementary Table 5**). Across both metrics, we observed peak alpha diversity in May, less than one-month post-release (**Figure 2**). The Shannon diversity and OTU richness of headstart lizard gut microbiomes in May was significantly different from all other months except June (**Supplementary Tables 4, 5**). Both metrics decreased in June and did not differ from any months in captivity by July (**Figure 2**; **Supplementary Tables 4, 5**).

We found significant differences between headstart and resident lizard gut microbiomes in May 2023 for both alpha diversity metrics: Shannon diversity (H = 14.84; p < 0.001) and OTU richness (H = 12.71; p < 0.001). When comparing headstart and resident lizard gut microbiomes in July 2023, two months post-release, we observed no significant differences for either Shannon diversity (H = 2.04; p = 0.153) or OTU richness (H = 0.003; p = 0.96; **Figure 2**; **Supplementary Table 6**).

To assess differences in non-gut microbial community diversity between the OKC Zoo and TAFB, we compared the alpha diversity of microbial communities recovered from diet and environment samples collected in each location. Environmental microbial community samples exhibited significant differences in Shannon diversity (H = 4.40; p<0.05) but not OTU richness (H =0.09, p = 0.76). Captive and wild diet microbial community samples did not differ in either Shannon diversity (H = 0.23; p = 0.63) or OTU richness (H = 1.07; p = 0.30; **Supplementary Table 6**).

To replicate the structure of our alpha diversity comparisons, we compared both beta diversity metrics between headstart and resident lizards in May and July 2023. During the month of May, both unweighted Unifrac and weighted Unifrac analysis detected significant differences in microbial community composition (p = 0.006 and p = 0.025, respective). No analyses showed significant differences between headstart and resident lizard gut microbiomes during the month of July (p = 0.176 and p = 0.107, respectively; **Supplementary Table 7**).

**Figure 3:**
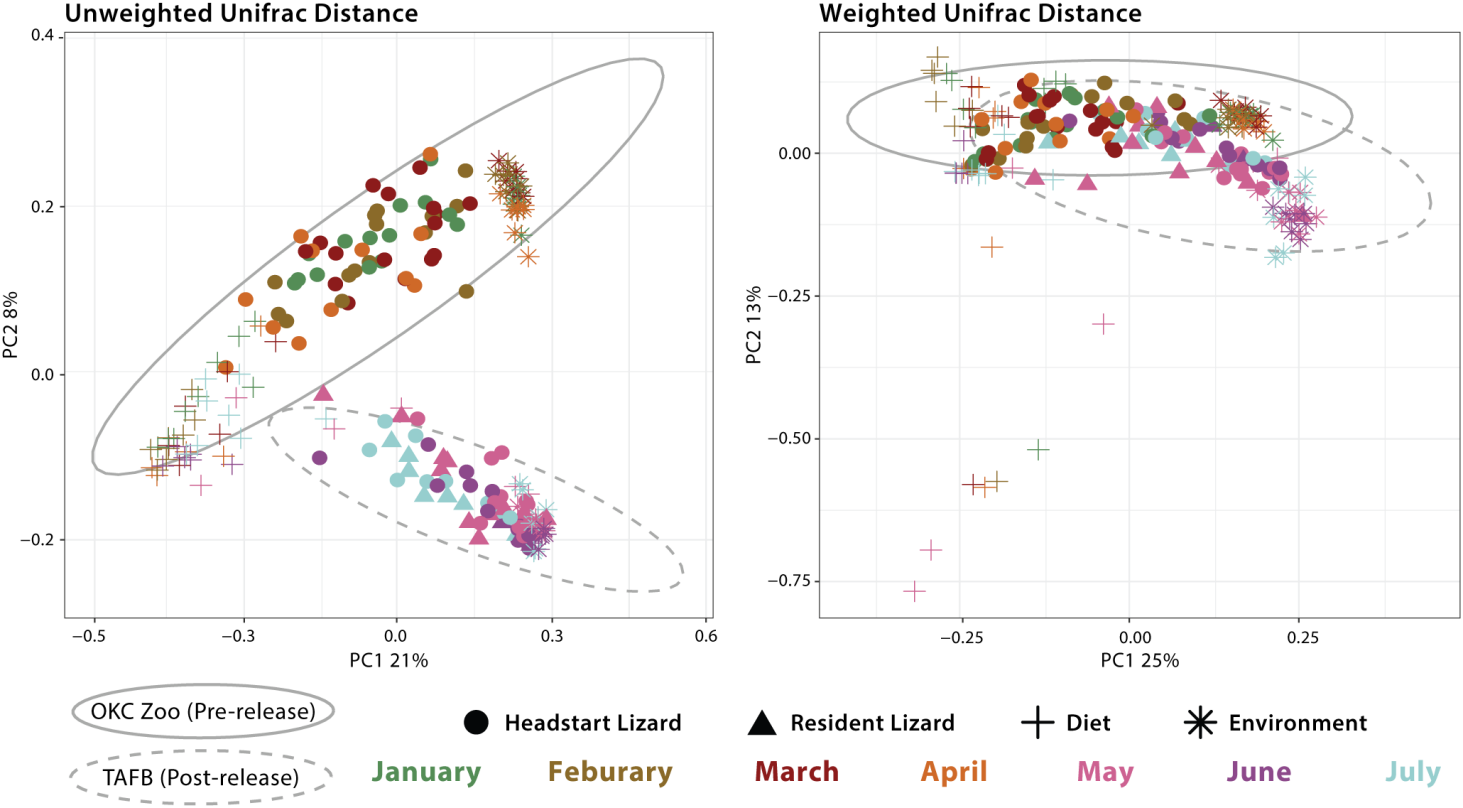
Principal Coordinates Analysis (PCoA) of all samples based on unweighted Unifrac and weighted Unifrac distance. Shapes represent different sample types: headstart lizard gut microbiome, resident lizard gut microbiome, dietary microbial community, and environmental microbial community. Colors correspond to month of sample collection. Gray ellipses demonstrate 95% confidence intervals for sample collection location (OKC Zoo or TAFB).

We also analyzed beta diversity (again unweighted and weighted Unifrac distances) among pairs of sampled microbial communities (headstart lizard, resident lizard, diet, environment). To best determine compositional differences among these diverse microbial communities, we grouped samples as follows: 1) headstart gut microbiome pre-release; 2) headstart gut microbiome post-release; 3) resident gut microbiome; 4) diet pre-release; 5) diet post-release; 6) environment pre-release; and 7) environment post-release. All pairwise comparisons resulted in significant differences (p<0.05) between these groups for both metrics (**Figure 3**; **Supplementary Tables 8, 9**).

**Figure 4:**
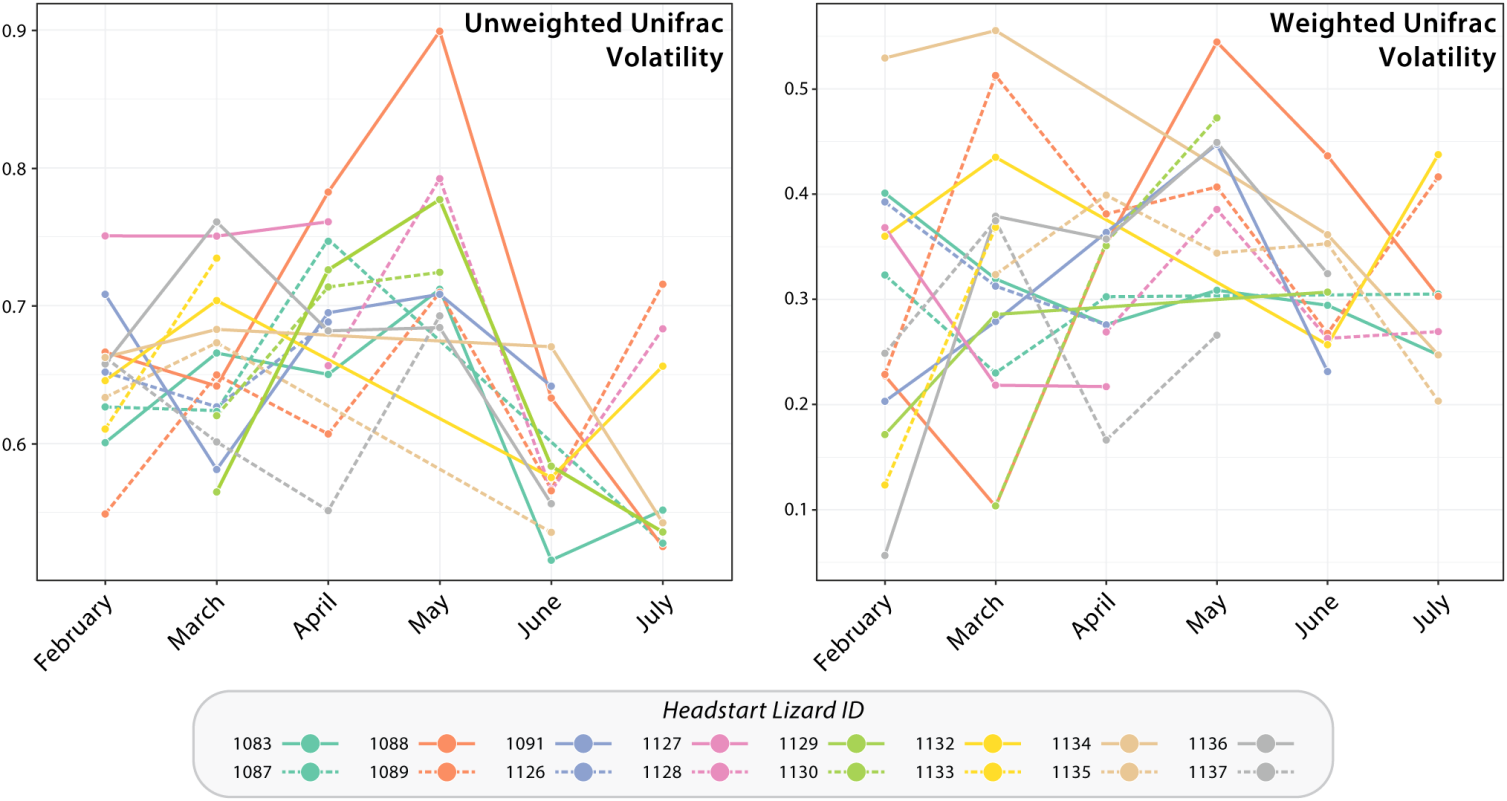
Volatility plots based upon unweighted and weighted Unifrac distance of headstart lizard gut microbiome samples from January to July 2023. Each line graph corresponds to a headstart lizard and displays the breadth of change in their successive gut microbiome samples. An upward slope indicates increased differentiation of the gut microbiome between time points, whereas a downward slope lower differentiation or less change in microbial community composition and structure.

Intra-individual monthly changes in headstart lizard gut microbiomes from January to July 2023 were visualized with a volatility plot (**Figure 4**). Our data visualization did support increased differentiation throughout the transition from captivity to the wild (i.e., from April to May), as demonstrated by an upward slope between these months (**Figure 4**). However, these data still suggest changes in individual gut microbiome community composition and structure during captivity. It is important to note that the poor sequence quality of some headstart lizard gut microbiome samples prevented having representative samples for every individual per month.

## 4 Discussion

Our study aimed to clarify how conservation translocation programs affect the host-associated microbiome. First, to better understand how diet and environment shape the host-associated microbiome, we examined extrinsic microbial communities observed across captivity and the wild. Next, we compared the post-release headstart gut microbiome to wild residents to investigate if periods of captivity had a lasting impact on the host-associated microbiome. Finally, we assessed longitudinal changes in the headstart lizard gut microbiome associated with their reintroduction to understand the impact of translocation practices. Overall, our study provides insight into the health and fitness of reintroduced animals. Conservation programs such as captive breeding and headstarting rarely incorporate post-release monitoring in any form, leading to a lack of knowledge surrounding the survivorship, fitness, and microbiome changes of headstarted/captive bred individuals post-release (Germano and Bishop, 2009; Brown et al., 2021). Monitoring the gut microbiome of released individuals could be one way to obtain information related to their health and fitness, especially when compared to wild residents.

### 4.1 Contributions of diet and environment to lizard gut microbiome in captivity and the wild

It is well-established that changes in the diet and environment of a host impact the structure and community diversity of the gut microbiome (Yuan et al., 2015; Jiang et al., 2017; West et al., 2019). However, it is much less clear if microbes from these sources are horizontally transmitted to the gut microbiome. Although it is plausible that a host consuming prey items or interacting with their habitat provides a pathway for microbes to colonize the gut, the core microbiome is likely determined by the metabolic needs of the host, which shift in response to dietary and environmental changes (Holmes et al., 2019). Other work on the squamate reptile gut microbiome provides mixed evidence for this source/sink relationship: a study on the gut microbiome of omnivorous lizards *Liolaemus parvus* and *Liolaemus ruibali* found little to no overlap with the invertebrate or soil microbiome, but almost 40% overlap with microbial communities on plant material in the same environment (Kohl et al., 2017).

Interestingly, in our study, we found little evidence that dietary and environmental microbiomes contribute significantly to the gut microbiome. Throughout captivity and the wild, these extrinsic microbial communities are compositionally distinct from the lizard gut microbiome (**Supplementary Table 8, 9**). This is also seen in our PCoA plots, where diet, environment, and gut samples form distinct clusters with little convergence (**Figure 4**). Furthermore, we identified unique shifts in the taxonomic composition of the headstart gut microbiome post-release unlike those associated with the diet and environment. Post-release, the headstart gut microbiome was uniformly enriched with Actinobacteriota (increase in average relative abundance from 15.06% in captivity to 32.63% in the wild) while the proportion of Proteobacteria (54.28% in captivity to 29.88% in the wild) and Firmicutes (18.89% in captivity to 8.42% in the wild) decreased (**Figure 1**; **Supplementary Table 2**). When comparing the captive and wild environment microbiome samples, we did observe a decrease in the average relative abundance of Firmicutes (18.66% in captivity to 2.18% in the wild), but the average relative abundance of Proteobacteria (30.69% in captivity to 32.27% in the wild) and Actinobacteriota (13.10% in captivity and 16.80% in the wild) was relatively (**Figure 1**; **Supplementary Table 2**). Meanwhile, in the prey item microbiomes, Actinobacteriota remained relatively consistent (2.48% in captivity and 3.90% in the wild), and we saw a decrease in the relative abundance of Firmicutes (41.13% in captivity to 4.93% in the wild) but a sharp increase in that of Proteobacteria (35.55% in captivity to 68.49% in the wild; **Figure 1**; **Supplementary Table 2**). These results suggest that the taxonomic composition of the headstart gut microbiome may not be directly associated with extrinsic microbial communities. Instead, the compositional shifts in the gut microbiome could be caused by other factors like increased interaction with conspecifics, or stem from extrinsic sources we did not include in our sampling, such as air or human handling.

Given that captivity presents a more stable and homogenous environment than the wild, it is plausible to assume that the extrinsic microbial communities would contain lower levels of alpha diversity (within-sample diversity). While we found that wild environmental microbiomes had higher Shannon diversity than those in captivity, there was no significant difference in OTU richness (**Supplementary Table 6**). We note that the substrate used in the captive enclosures at the OKC Zoo contained some locally sourced soil, which could contribute to similar alpha diversity across captive and wild environmental microbiomes. We also found no significant differences in the alpha diversity of captive and wild prey item microbiomes (**Supplementary Table 6**). Previous studies on vertebrates have similarly identified no difference in alpha diversity across captive and wild populations (Scupham et al., 2008; Campos et al., 2018; Tong et al., 2019; Diaz and Reese, 2021). Additionally, a past study on the Comal Springs riffle beetle (*Heterelmis comalensis*) found that the microbiome of captive beetles was more diverse than wild beetles (Mays et al., 2021). These results contrast the common notion that wild microbial communities are less homogenized than captive ones (Dallas and Warne, 2022).

### 4.2 Texas horned lizard gut microbiome composition

Our study provides novel insight into the gut microbiome of squamate reptiles, an overall understudied taxa within the literature (Colston and Jackson, 2016). Most host-associated microbiome work focuses on humans or mammals, while a limited number of studies feature reptiles as the focal taxa (Siddiqui et al., 2022). Further, even fewer studies have investigated the microbiomes of threatened reptilian species (Colston, 2017), leaving major gaps in our knowledge of the microbial community composition and structure of vulnerable reptile taxa.

Previous studies have suggested that Bacteroidota, Proteobacteria, and Firmicutes are core phyla of the reptile gut microbiome (Siddiqui et al., 2022). We identified these phyla in all of our lizard samples, although at varying relative abundances. Proteobacteria had the highest relative abundance in pre-release headstarts when compared to post-release headstarts and residents (average relative abundance = 54.28%; 29.88%; and 31.45%, respectively; **Supplementary Table 2**). Similarly, the phylum Firmicutes was present at a greater abundance in pre-release headstarts compared to the other groups (18.89%; 8.42%; and 9.27% respectively). Meanwhile, Bacteroidota was only present at a relative abundance of 3.51% to 5.82% across lizard sample groups. Beyond these three phyla, we also observed Actinobacteriota at a high average relative abundance (>15%) across all lizard samples (**Supplementary Table 2**). Other studies have also identified Actinobacteriota as a prominent phylum in lizard gut microbiomes (Tang et al., 2022; Vasconcelos et al., 2023).

Preliminary data on the gut microbiome of the first *P. cornutum* lizard cohort released to the wild in 2021 was primarily composed of Bacteroidota and Firmicutes (both present at average relative abundances >25%) across pre-release, post-release, and wild individuals (S. Eliades personal communication). The lower average relative abundance of Bacteroidota within our samples may be due to sample collection differences. We collected cloacal swabs as a proxy for sampling the gut microbiome, whereas the previous study used fecal samples. Work on the gut microbiome of *Sceloporus virgatus* lizards found that fecal samples contained higher proportions of Firmicutes and Bacteroidetes, whereas cloacal swabs contained a higher proportion of Proteobacteria (Bunker et al., 2022).

### 4.3 Temporal Texas horned lizard gut microbiome dynamics across captive and wild environments

Previous research on wildlife host-associated microbiomes has established that captive and wild populations differ in community composition and structure (Trevelline et al., 2019). However, much less is understood regarding how transitions between captive and wild settings may affect the host-associated microbiome over time. Most studies demonstrate that the gut microbiome of translocated individuals will eventually restructure to be indistinguishable from wild counterparts (Chong et al., 2019; Schmidt et al., 2019; Yao et al., 2019; Korpita et al., 2023). However, the length of time for the gut microbiome to restructure varies widely; studies on the Tasmanian devil (*Sarcophilus harrisii*), deer mouse (*Peromyscus maniculatus*), and boreal toad (*Anaxyrus boreas*) suggest a restructuring period of two to four weeks, while research on the giant panda (*Ailuropoda melanoleuca*) suggests up to a year was needed for the gut microbiome to lose signatures of captivity (Chong et al., 2019; Schmidt et al., 2019; Yao et al., 2019; Korpita et al., 2023). Differences in life history strategy and ecology among these species may contribute to variation in the gut microbiome restructuring period.

Within squamate reptiles, a study on the critically endangered Fijian crested iguana (*Brachylophus vitiensis*) found that the gut microbiomes of translocated individuals took two months to be indistinguishable from the wild counterparts (Eliades et al., 2021). Similarly, our results demonstrate that the gut microbiomes of reintroduced lizards resembled those of wild ones within two months of release. (**Figure 2**). Within one month, traces of captivity lingered: headstart and resident lizards displayed significant differences in both alpha and beta diversity indices in May (**Figures 2**, **3**). However, when headstart and resident lizards were sampled again in July (two months post-release), no diversity metrics differed among these populations (**Figures 2**, **3**).

It is interesting to note the peak in alpha diversity of the headstart lizard gut microbiome immediately following their translocation to WR3 in May (**Figure 2**). One explanation could be that the gut microbiome of headstart lizards enters a hyper-diverse state distinct from both their own previous gut microbial communities displayed in captivity and those of their wild counterparts. Linear mixed models demonstrated that the alpha diversity of the headstart lizard gut microbiome in May is significantly higher than all other months except June (**Figure 2**; **Supplementary Tables 4, 5**), and Kruskal-Wallis tests further supported this conclusion (**Figure 2**; **Supplementary Table 6**). Although studies on mammalian and amphibian taxa have not noted this pattern (Chong et al., 2019; Schmidt et al., 2019; Yao et al., 2019; Korpita et al., 2023), a study on the Fijian crested iguana (*Brachylophus vitiensis*) also found the alpha diversity of post-release individuals to be higher than wild counterparts (Eliades et al., 2021). During this period, beyond adjusting to a new diet and habitat, the headstart lizards are experiencing other novel environmental factors like fluctuating temperatures and rainfall which could all contribute to variation in gut microbiome diversity (Moeller et al., 2020; Williams et al., 2022). Another factor may be newfound social interactions. In captivity, the headstart lizards were housed individually, and then placed together for the first time in the soft-release pen. Studies on crocodile lizards have also noted increased community richness associated with cohabitation (Tang et al., 2020). Lastly, the observed peak in alpha diversity may be due to the transitory state of the gut microbiome; microbes found predominantly in captivity may remain, while concurrently, those found in the wild are beginning to colonize the gut microbiome, leading to a high level of community diversity. Future studies may more closely examine gut microbiome assembly throughout this transitional period.

### 4.4 Conservation applications

Given the relationship between the gut microbiome and animal health, it is important for conservation practitioners to consider how translocations between captive and wild settings impact animal fitness. Although our results demonstrate that the gut microbiome community composition and structure of translocated individuals does shift to match wild counterparts within two months post-release (**Figures 2**, **3**), the in-between period of restructuring could have fitness consequences. Our beta diversity volatility plots demonstrate that the gut microbiomes of some headstart lizards display increased change in community composition and membership from April to May, when they were reintroduced (**Figure 4**). Additionally, this period coincides with a peak in headstart lizard alpha diversity, suggesting that new bacterial species are colonizing the microbiome (**Figure 2**). Both findings indicate that the first month post-release could be a period of instability for the gut microbiome. A destabilized gut microbiome may derail beneficial physiological functions for the host, alter behavior, or even hinder reproductive output (Desbonnet et al., 2014; Zaneveld et al., 2017; Comizzoli et al., 2021; Barathan et al., 2024; Sonnega and Sheriff, 2024). Future studies may more closely examine how gut microbiome variation and destabilization correlates with changes in host spatial movements or behavior post-release.

Conservation practitioners may consider implementing soft-releases practices to allow host-associated microbiomes of released individuals to restabilize and adjust to the new environment. Translocation soft-release practices involve constructing enclosures in the new habitat to allow for adaptation without the risk of predation (Resende et al., 2021). In this case, if translocated individuals are at a fitness disadvantage due to the restructuring of the gut microbial community, a soft release would also better support their survivorship. In our study, the headstart lizards were placed in an outdoor enclosure for a three-week soft release period upon their release to TAFB in May 2023, which happen to coincide with the period of gut microbiome restructuring.

It is important to note that our study utilized 16S rRNA amplicon sequencing, which only provides insight into taxonomic community diversity. Without metagenomic sequencing, it is not clear if the restructuring in community composition and membership also indicates a change in physiological functions provided to the host. Future studies should consider utilizing whole-genome or shotgun metagenomic techniques to better investigate the metabolic capabilities and functions of microbes within and across these communities. Although our study found evidence of a taxonomic restructuring period of the gut microbiome community, future work could investigate if this also corresponds with a functional restructuring.

Overall, our work provides insight into longitudinal microbial community diversity dynamics associated with host transitions from captive to wild environments. It is clear the animal gut microbiome is sensitive to environmental change and may take a variable time period to restabilize after translocation. Given the widespread anthropogenic environmental changes animal populations are facing globally, and the physiological importance of the gut microbiome for host health, it is essential to better understand microbial community dynamics and its relationship to host fitness and survivorship.

## Data Availability

The datasets presented in this study can be found in online repositories. The names of the repository/repositories and accession number(s) can be found below: https://www.ncbi.nlm.nih.gov/, PRJNA1218705.
