# Supplementary material for: Comparative assessment of Texas horned lizard (Phrynosoma cornutum) gut microbiome diversity and composition throughout transition from captivity to wild
Source: Front Microbiomes. 2025 Jun 18;4:1601442. doi: 10.3389/frmbi.2025.1601442 (PMC12993635; doi:10.3389/frmbi.2025.1601442)
Supplement: Supplementary file 1 [file SupplementaryFile1.docx]

Supplementary Material

# Supplementary figure captions

**Supplementary Figure 1.** Taxonomic barplots displaying recovered versus expected composition of microbial community and microbial community DNA standards.

**Supplementary Figure 2.** Alpha rarefaction curves based on Shannon Diversity Index. Each curve represents a unique sample. The dataset was rarified to a sequence count of 500 for downstream analysis.

**Supplementary Figure 3.** Alpha rarefaction curve based on OTU Richness. Each curve represents a unique sample. The dataset was rarified to a sequence count of 500 for downstream analysis.
