# Supplementary figures and images for: Comparative assessment of Texas horned lizard (Phrynosoma cornutum) gut microbiome diversity and composition throughout transition from captivity to wild
Source: Front Microbiomes. 2025 Jun 18;4:1601442. doi: 10.3389/frmbi.2025.1601442 (PMC12993635; doi:10.3389/frmbi.2025.1601442)

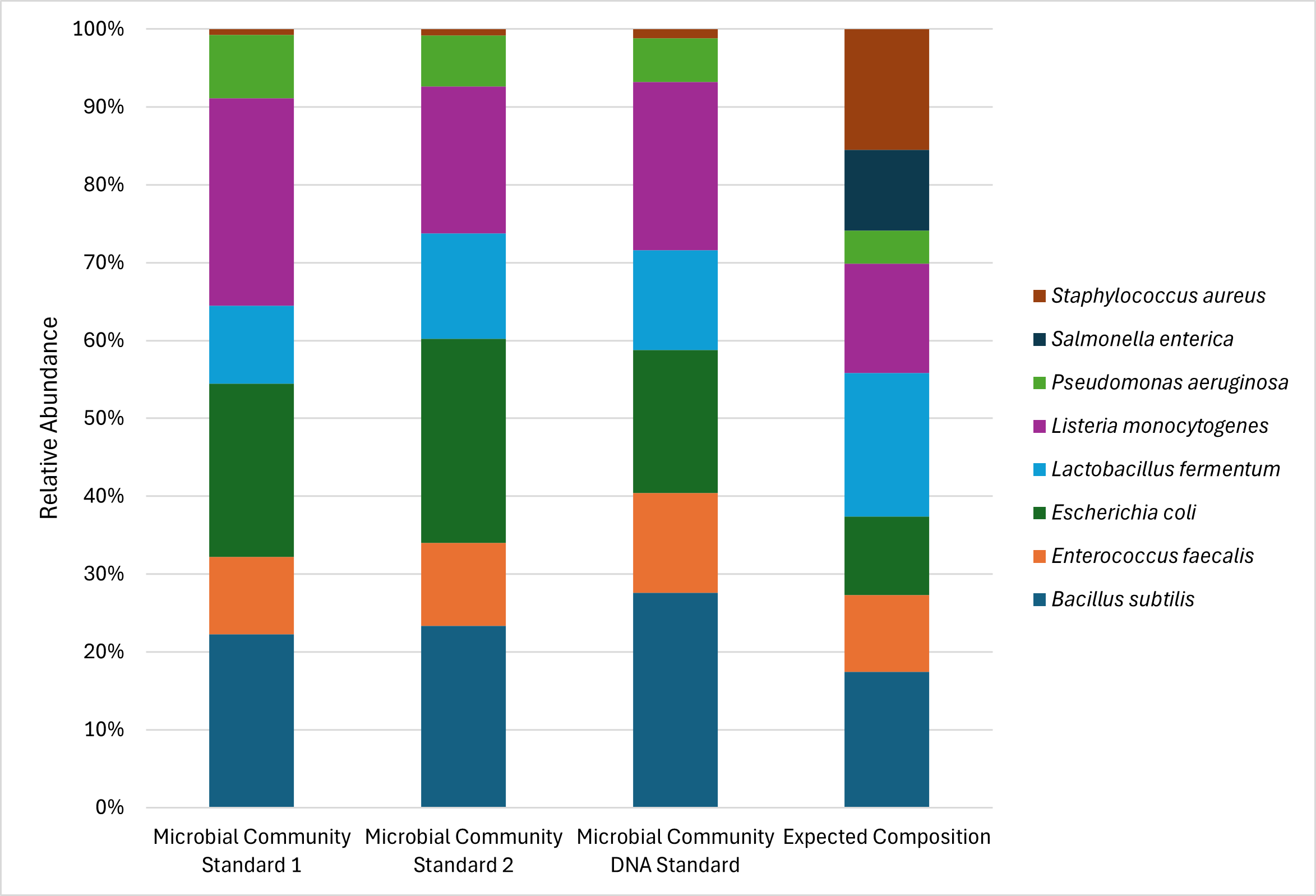

Supplement: Supplementary file 2 [file Image1.tiff]

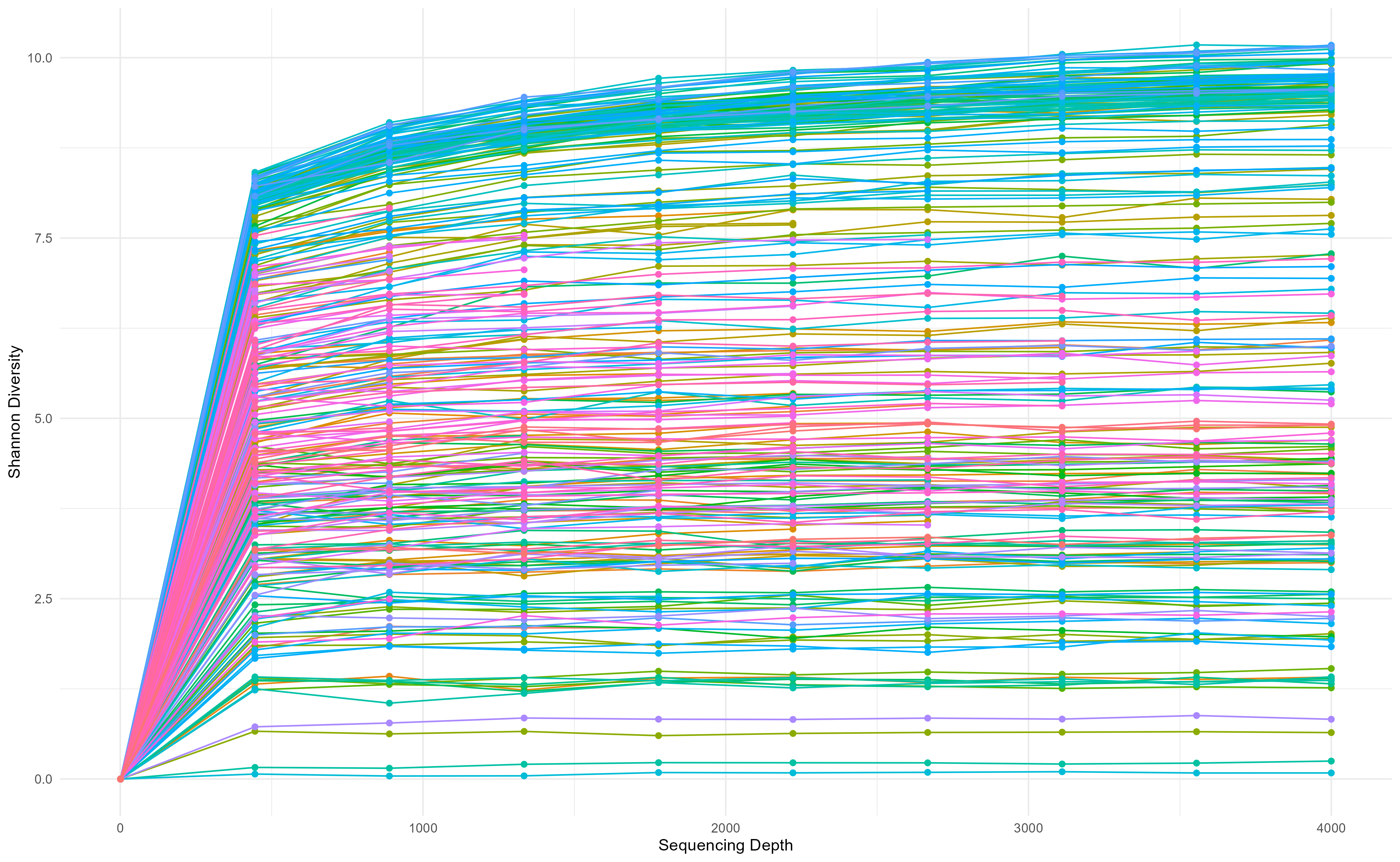

Supplement: Supplementary file 3 [file Image2.tiff]

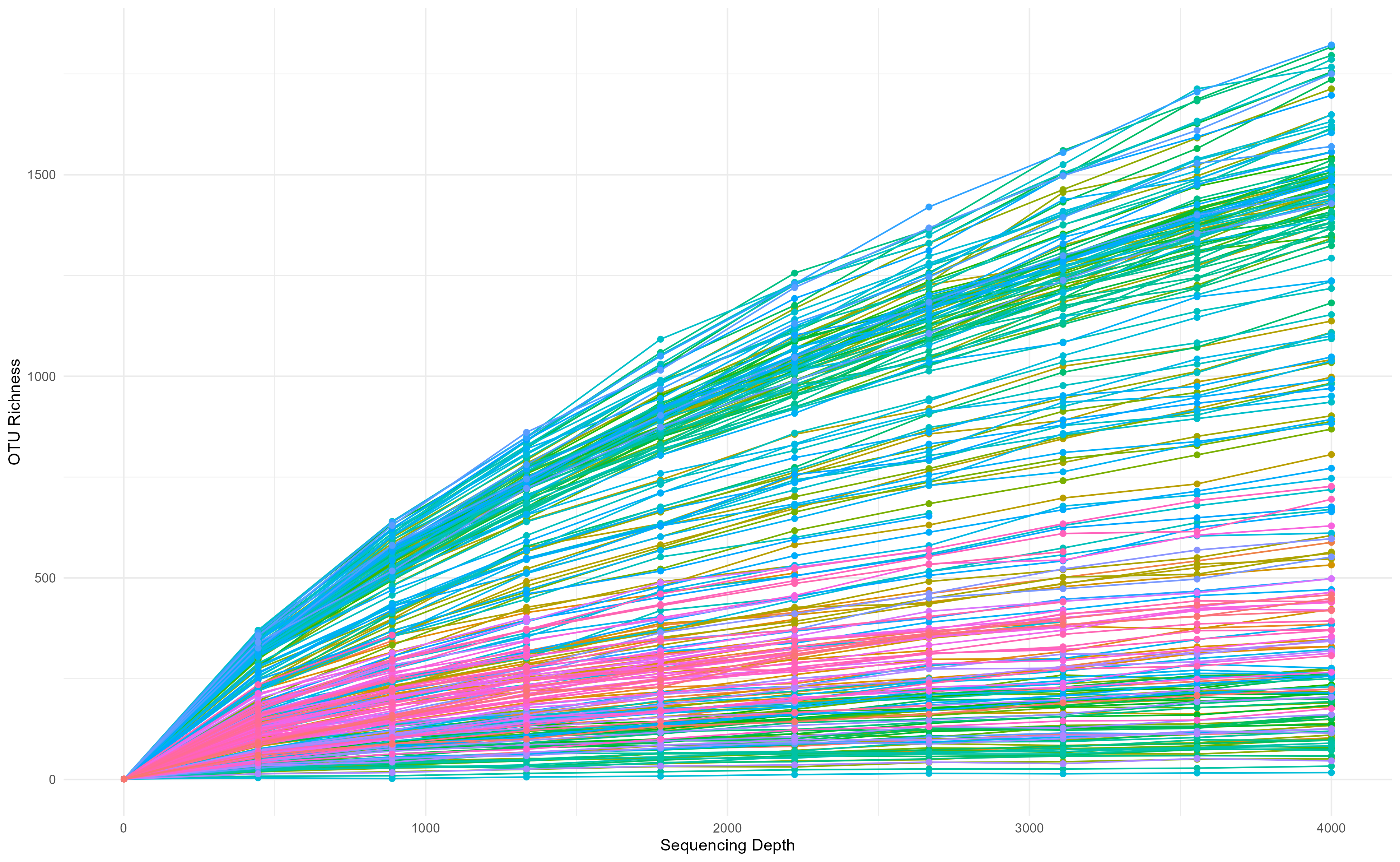

Supplement: Supplementary file 4 [file Image3.tiff]
